# Detection of Human Impacts by an Adaptive Energy-Based Anisotropic Algorithm

**DOI:** 10.3390/ijerph10104767

**Published:** 2013-10-10

**Authors:** Manuel Prado-Velasco, Rafael Ortiz Marín, Gloria del Rio Cidoncha

**Affiliations:** Multilevel Modeling and Emerging Technologies in Bioengineering (M2TB), University of Seville, Escuela Superior de Ingenieros, C. de los Descubrimientos s/n, Sevilla 41092, Spain; E-Mails: rafortiz@us.es (R.O.M.); cidoncha@us.es (G.R.C.)

**Keywords:** adaptive algorithm, energy-based impact detection, unsupervised learning technique, telehealth services, distributed signal processing, smart sensor

## Abstract

Boosted by health consequences and the cost of falls in the elderly, this work develops and tests a novel algorithm and methodology to detect human impacts that will act as triggers of a two-layer fall monitor. The two main requirements demanded by socio-healthcare providers—unobtrusiveness and reliability—defined the objectives of the research. We have demonstrated that a very agile, adaptive, and energy-based anisotropic algorithm can provide 100% sensitivity and 78% specificity, in the task of detecting impacts under demanding laboratory conditions. The algorithm works together with an unsupervised real-time learning technique that addresses the adaptive capability, and this is also presented. The work demonstrates the robustness and reliability of our new algorithm, which will be the basis of a smart falling monitor. This is shown in this work to underline the relevance of the results.

## 1. Introduction

The epidemiology of falls in the elderly population has been widely studied over the last three decades [1,2,3,4]. Falls in this population have severe health consequences, including bone fractures, soft and connective tissue damage, and head injuries [2,5]. They are also one of the primary causes of death among elderly people [6].

Many studies point to the importance of falls in the economic burden of the health-care system [7]. As a reference, in 2000, direct medical costs among US adults older than 65 years were $0.2 billion dollars for fatal injuries and $19 billion dollars for non-fatal injuries associated with falls, [8]. Medical expenditures for women (58% of the older adult population) was 2–3 times higher than for men. The cost of fall injuries in the US has been estimated at $32.4 billion for 2020 [2]. Similar trends and health consequences have been claimed in other countries [7]. As a consequence, research on fall detection and prevention for the elderly has been included in the European Framework Programs and National Scientific Programs directives.

Boosted by these results, as well as by the needs of sociohealthcare providers that demand solutions to detect and prevent falls, researchers and technicians have been working on the development of falling monitors since the 1990s [9]. Ideally, these systems could also give clues and indicators that assist multifactorial programs on fall prevention [3].

Two main requirements are demanded by users and caregivers: *unobtrusiveness* and *reliability*. The first is related to discretion, size, and ergonomics, whereas the second is associated with the efficacy to detect falls. This is, in turn, related to the signal processing technology used, which should provide a near 100% sensitivity (all falls are detected), and a very high value of specificity (low number of false falling alarms or positives).

Different systems have been proposed for automatic fall detection, with varying degrees of success, using sensors embedded in the home environment, wearable sensors with real-time processing, or ambulatory systems [10]. A recent worthwhile example is given by an algorithm to detect near falls [11]. These systems use mainly inertial micro-electromechanical systems (MEMS), such as accelerometers and gyroscopes, owing to their very low power consumption and size, and the ability to manage local digital communications to a host processor.

Among existing relevant commercial falls detection systems we must highlight the fall detector from Tunstall^TM^, evolved from [12], the Philips^TM^ Lifeline with AutoAlert for detecting falls [13], the Zenio^TM^ fall detector [14], and the fall detection reporting system from Fatronik-Tecnalia^TM^ [15]. All of these are founded on similar inertial MEMS. Although these companies claim their systems’ unobtrusiveness and reliability, it is a fact that they have not achieved wide diffusion among sociohealthcare providers yet. Moreover, their claims are not supported by published laboratory or clinical studies.

This poor scenario can be justified on the basis of the high rate of false alarms, together with the poor ergonomics and discretion of current systems [16]. We analyzed this situation in earlier studies [17,18], concluding that barriers to a wide deployment of fall detectors are a result of the difficulty of combining high reliability with low obtrusiveness, together with the rigid definition of falls performed by most real-time systems. Reliability requires a good processing capability, whereas discretion compels a very small size and low energy consumption. There are other related factors, such as the availability to adapt the sensor fixation and placement, according to the needs and psychological preferences of the users.

Our preliminary studies proposed that an adaptive (continuous personalization) system, based on a distributed architecture, could reach both objectives [17,19], and even generalize the definition of a fall [18]. Following the approach presented in [20], we consider two types of Physical Risk Events (PREs) that a 2-layer falling monitor should detect: impact, and non-impact based. Body impacts trigger a subsequent analysis of accelerations in the second processing layer (analysis of subject activity), to decide if the subject has suffered an impact-based PRE. Non-impact based PREs are detected by periodic polling. This work presents a very light algorithm to detect body impacts, and the evolution of a previous one [16], which is able to adapt the thresholds to a subject in a continuous and unsupervised way, thus optimizing its performance. The existence, robustness and reachability of the optimum region of thresholds is carefully addressed. This is the basis of the non-impact based PREs of a novel smart monitor, whose conceptual design is outlined to clarify the whole scenario. Other issues of this monitor, such as hardware design and the second processing layer, exceed the scope of this work.

## 2. Methods and Materials

### 2.1. Functional Specifications of a Reliable Falling Monitor

There are two fundamental functional specifications that a PRE detector should meet, to achieve market diffusion [16,18]:

1. It must guarantee a sensitivity value near 100%, with a very low rate of false alarms (high specificity), in all situations and environments where the supervised subject lives.
2. The system must be emotionally accepted by target subjects. A well-known derived requirement is the unobtrusive character of the system [21].

As a consequence of earlier results [17], we hypothesize that these fundamental specifications can be reached by a wearable accelerometric monitor, founded on the following methodological issues:

1. Personalization. The monitor should be customized to the subject under surveillance. The changing nature of human behavior and health suggests that the system must evolve with the subject. We call this an “adaptive monitor” since it is able to follow the associated subject in a continuous way.
2. Physical Risk Events (PRE) detector. Falls can be classified in impact-based PREs and non-impact-based PREs. Body impacts must trigger the analysis of activity around the event, to detect impact-based PREs.
3. Computational architecture functionally partitioned. A distributed processing architecture, with a division of modules on account of functional tasks, should be a solution to the current limitations on the processing capacity of unobtrusive smart monitors.
4. Attention to personal preferences and needs. This requirement agrees with the necessity to allow changes in the position of the sensor, because of health conditions (e.g., dermatitis).

These four hypotheses are linked. For example, the ability of the monitor to follow the changing behavior of the subject (hypothesis 1) compels the design of adaptive processing algorithms, which in turn facilitates the attention to changes in the place of fixation of the accelerometric sensor (hypothesis 4). These changes respond both to emotional parameters (preference) and health restrictions. For example, in the case of using a sticking plaster solution as a disposable bag for the smart sensor, the skin position must be changed every 4–6 days, as a function of the skin state of the subject, to avoid inflammation.

This work addresses the analysis of these hypotheses, with the aim of developing a novel impact detection algorithm that can be used subsequently to monitor impact-based PREs. We start from an earlier design [22,23], which was tested in a laboratory in a preliminary study [17]. Results from that work suggested that personalization increases the area under the curve of Receiver Operator Characteristic (ROC) of an impact detection algorithm, implemented in the smart accelerometric sensor of a movement monitor. However, those results were preliminary. In addition, they did not take into account the influence of the number of experiments in the ROC, nor the existence and reachability of the region of the algorithm’s parameters, that can actually be associated with an optimum region in the ROC space, in terms of sensitivity and specificity.

### 2.2. Laboratory Study

Here, we extend the analysis performed in an earlier study [17], with data obtained using the same intelligent accelerometer sensor, with the aim of facilitating the comparison of results. The hardware and signal processing details are described in the following subsections. The subsequent analysis tries to quantify the ability of a smart adaptive algorithm, based on accelerometric signals in detecting body impacts of a subject under surveillance.

**Figure 1:**
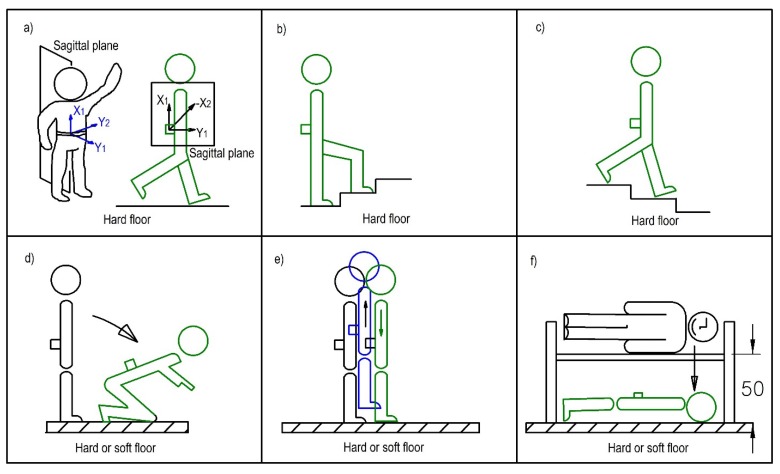
Set of physical activities performed in the laboratory study. Upper row shows the five types of non-impact activities: walking exercises (a), going upstairs (b) going downstairs (c). The same diagram shows the accelerometer axes. Axes x_1_, y_1_, and x_2_ pertain to the sagittal plane (near central plane), according to the position of sensor, and x_2_ follows the bisectrix, and is directed towards the origin. System x_1_, y_1_, y_2_ is orthogonal. The lower row depicts the 6 impact activities (three per type of floor): falling to the knees (d), vertical jump (e), and horizontal falling (f).

We define a set of physical activities, divided into normal (non-impact) and shock activities (impact), identified in Figure 1. Normal activities are defined as slow walking, normal walking, fast walking, going upstairs, and going downstairs (all of them on hard floor), whereas shock activities are defined as vertical jump, falling to the knees, and horizontal falling from a bench (all of them on hard and soft floors). The bench’s height was 50 cm. The smart sensor was worn on the back near the sacrum. We used four accelerometric axes (vertical and horizontal into a sagittal plane; a bisectrix to them; and perpendicular to sagittal plane). In opposition to normal activities, shock ones were performed on hard and soft floors. Therefore, each set of activities to be performed has 11 different exercises (five normal plus six shock activities).

The study was carried out using 13 healthy and young pregraduate volunteers, who gave informed consent. One subject was selected randomly among them to repeat the set of 11 activities eight times. These subjects (six males, seven females) ranged in age from 23 to 31 years (25.9 ± 2.2), weighed from 49 to 89 kg (67.2 ± 12.76), and were 1.54 to 1.83 m (1.68 ± 0.08) tall. 

We did not recruit older adults for the study, even though they could perform non-impact activities properly, because they cannot be compared against impact activities under a personalized and adaptive approach.

### 2.3. Accelerometer Sensor Hardware Used in the Study

The study was performed by means of a smart accelerometer sensor prototype, called an intelligent accelerometer unit (IAU) [22]. This device pertains to an earlier patented falling monitor [24], and it comprises two MEMS accelerometers (ADXL202E), mounted in an arrangement to provide the four accelerometric directions. A Microchip^TM^ PIC16LC66 8-bit CMOS microcontroller was used to sample and demodulate the duty cycle-modulated (DCM) output of both ADXL202E devices, as well as to execute the algorithm presented in the following subsection.

### 2.4. Algorithms for Impact-Based PRE Detection: Definition and Evaluation

We started with an isotropic impact-detection algorithm, based on the following equations [17]:

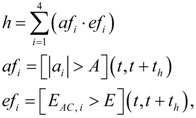
(1)
where the output variable, *h*, gives a prediction of positive impact when *h* = 1 (true). Concerning this equation, *af_i_* and *ef_i_* are binary variables that are activated (true) during the interval (*t*, *t* + *t_h_*), starting from the instant *t*, where thresholds *A* and *E*, defined in the two last equations, are surpassed. Binary variables are defined for each measurement axis, *i*. Sum and multiplication operators in the first equation refer to OR and AND logical conditions. Variables |*a_i_*| and *E_AC,i_* are the absolute value of the acceleration signal and the energy of the acceleration signal, after removing the DC component, in the *i*-axis. Because of computational cost reduction, squared values of accelerations in Energies were substituted by absolute values [23], giving the following equation for *E_AC,i_*:

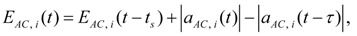
(2)


where τ is the width of the temporal window to which the energy is referred (accumulated), *t_s_* is the sampling time for accelerations, and *a_AC,i_* is the acceleration signal filtered by a DC suppressor. The latter is given by the following FIR (finite impulse response) filter in its z-transform:

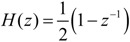
(3)


This low-order FIR filter was selected because of the very low computation load and memory required [25]. As z^−1^ is a *t_s_*-shift, it can be written *a_AC,i_*(*t*) = (*a_i_*(*t*) − *a_i_*(*t* − *t_s_*))/2.

The execution of the isotropic algorithm in the IAU’s prototype used in this work, with *t_s_* = 27.5 ms, τ = 660 ms, and *t_h_* = 1.76 s, required that PIC16LC66 was active for nearly 35% of real time [17]. This value includes the decoding of PWM accelerations inputs from four channels of two ADXL202.

The isotropic algorithm was studied previously, and provided successful results [17], but the analysis was preliminary. It has now been completed and extended to form a novel anisotropic algorithm in this work. The anisotropic algorithm was derived from the isotropic one, converting scalar thresholds into vectors, whose components, *A_i_* and *E_i_*, are associated with the measurement axes.

The accelerations were measured during the laboratory study, with the values of *t_s_*, τ, and *t_h_*, as previously indicated. The value of *t_s_* is justified because the bandwidth of human activities measured at the waist is in the 5–20 Hz bandwidth [26]. We selected a sampling frequency 1/*t_s_* = 36.4 S/s. The value of τ was taken around the smallest period that characterizes daily physical activities. The analysis verified the low sensitivity to this τ value, in agreement with earlier works [23]. Finally, the *t_h_* value takes into account the fact that maximums of energy and acceleration amplitude do not necessarily occur at the same instant. It allows a separation between these events around 2.5τ [23].

We have used the ROC space to analyze the goodness of the impact detection algorithms presented. We define *TP* as the number of true positives (correctly detected), *FP* as the number of false positives (incorrectly detected), *P* as the number of true positives, and *N* as the number of true negatives. Then the sensitivity of the algorithm is the true positive rate *tpr* = *TP/P* (per unit), and the specificity is 1 − *fpr* (per unit), where *fpr = FP/N* is the false positive rate.

In the case of the isotropic algorithm, we transform the parameter space, given by the 2 tuple (*A*, *E*), to the ROC space, given by the 2 tuple (*fpr*, *tpr*). In the case of the anisotropic algorithm with 3 axes, the parameter space is given by the 6 tuple (*A_i_*, *E_i_*). We used a smart sweep of the parameter space to obtain pairs (*fpr*, *tpr*) associated with each space point (threshold), by comparing the impact prediction with the type of activity (impact or non-impact) [27]. This method allows the evaluation of the algorithms’ reliability, by means of the area under the curve (AUC) in the ROC space, which gives the probability that the algorithm distinguishes negative (non-impact) from positive (impact) activities properly.

We have defined two scenarios to evaluate the goodness of algorithms. In the first one, algorithms were applied to the activities performed by the 12 subjects with one set each. Therefore, any point in the ROC space was obtained by computing *tpr* and *fpr* for 132 (12 × 11) experimental activities. This scenario provides results concerning the non-personalized performance of algorithms (*i.e*., thresholds are equal for all the subjects). In the second scenario, algorithms were computed over the 88 experimental activities performed by the subject with eight sets (8 × 11), providing results concerning the personalized performance of algorithms (*i.e*., thresholds are applied only to one subject).

In order to compare the ROC spaces obtained in the two scenarios (non-personalized *versus* personalized), the activities of the non-personalized scenario were taken in groups of eight subjects, selected randomly (*i.e*., eight × 11 activities) and averaged. We compared the average AUC of the non-personalized scenario with the AUC of the personalized scenario, for 88 (eight × 11) activities.

The optimal region of parameters, R_opt_, was defined by the set of points in the parameter’s space, such that their associated empirical ROC region verifies *tpr* = 1, and *fpr* < α, where α is the maximum value of false positive rate allowed. We researched the influence of α in the existence and robustness of R_opt_, in the personalized *versus* non-personalized form of the algorithm.

The analysis of the sensitivity of *fpr* and *tpr* to the variation of parameters inside, and at the boundary of R_opt_ provided the basis for the reduction of complexity of the algorithm given by Equation (1).

A subsequent analysis of the average energy per type of activity in the personalized algorithm supports the strategy to design a low-cost continuous learning technique, able to find parameters inside R_opt_. This technique provides the adaptive feature of the impact-detection algorithm.

The effect of withdrawing an accelerometric axis, keeping an orthogonal 3-axis measurement system, is also analyzed. Experimental results will focus on the comparison of the performance of the 4-axes isotropic algorithm against the 3-axis anisotropic algorithm.

### 2.5. Smart Monitor Sketch

The experimental outcomes obtained from the analysis of the new impact-detection algorithm, executed by a smart sensor, are the basis of the impact-based PREs, detected by a novel adaptive falling monitor. We do not present the hardware design nor the second processing layer of the monitor here, because it exceeds the scope of the paper. Nevertheless, the conceptual design is shown, including a block diagram with the main elements of the monitor, where the sensor’s algorithm works, since this is important for understanding the study.

## 3. Results

The analysis of the impact detection algorithm involved the following stages:

1. *Personalization*. It is shown that personalization improves the sensitivity and specificity of the isotropic algorithm, which can be considered a dichotomic classifier of activities (impact, no impact).
2. *Optimal parameter region*. It is shown that there is an optimal region in the parameter space of the algorithm, R_opt_, defined by a sensitivity of 100%, and a high specificity (low rate of false positives), which is robust.
3. *Functional partition*. The analysis of the efficiency of the algorithm inside R_opt_ will support the functional partition of the computational architecture of the monitor.
4. *Reachability*. It is shown that R_opt_ is reachable by means of an unsupervised continuous learning technique, with very low computational load, which provides the adaptive feature of the algorithm.
5. *Anisotropic Algorithm*. We verified that the performance of the 3-axis anisotropic algorithm surpasses that of the isotropic algorithm, although it keeps the remaining properties.

These points directly support hypotheses 1 and 3 (Methods section), concerning personalization and functional partition, respectively, and indirectly support hypotheses 2 and 4, because they allow the design of an unobtrusive wearable monitor of PREs.

### 3.1. Stage I. Personalization

**Figure 2:**
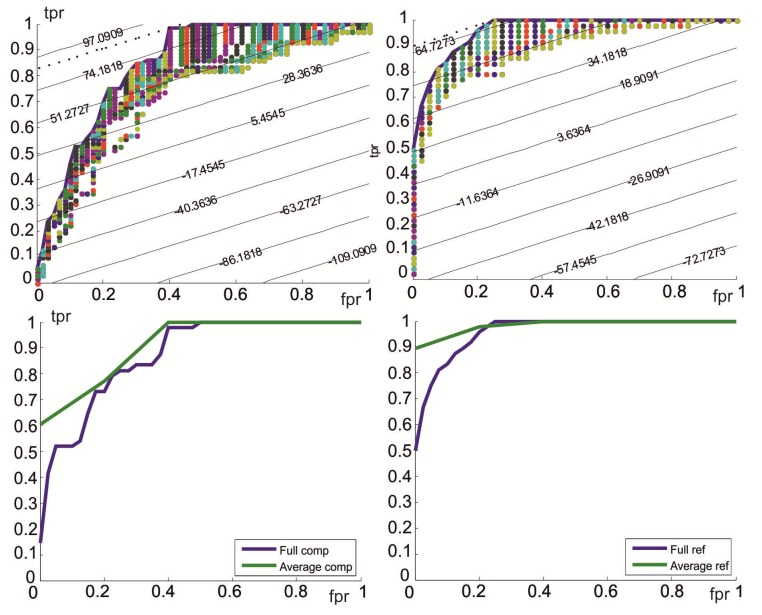
Top row: enveloping curves of ROC points associated with impact detection in the isotropic algorithm (Equation (1) with 4 axes) for the study carried out using 12 subjects with 1 set (**left**), and over the subject with 8 sets of activities (**right**). Bottom row: average of enveloping curves of ROC points obtained for each set (AUC_avg_) against the full ROC for all activities. Left: the full ROC is presented for a representative combination of 8 sets from the study with 12 subjects with 1 set. Right: the full ROC refers to the total 8 sets from the study over 1 subject with 8 sets.

The values of the area under the ROC enveloping curve associated with the isotropic algorithm (Equation (1)) for four axes were 0.8463 and 0.9609, for all the activities performed over the 12 subjects with one set (non-personalized), and the subject with eight sets (personalized), respectively. The enveloping curves are shown in Figure 2, together with the ROC points obtained for each 2-tuple (*A*, *E*) in the parameter space.

Although this result completes and supports previous conclusions about the superiority of personalized against non-personalized forms of the isotropic algorithm [17], it is necessary to withdraw the influence of the number of activities on AUC, as well as the potential influence of the manner of performing the physical activities by the subjects.

We selected 10 combinations of eight subjects randomly from the 12 subjects of the non-personalized experiment (88 activities in each combination), taking the mean of the 10 AUC values obtained (one for each combination). This mean value of AUC was equal to 0.8524, which can be compared to the AUC for the 8-set study (88 activities), equal to 0.9609. The difference of AUCs 0.9609 – 0.8524 = 0.1085 confirms clearly the advantage of personalization, after removing the influence of the number of activities.

To remove the influence of the subject, it is considered that the AUC value reaches a maximum when it is computed for a unique subject with only one set of activities (this the simplest controlled set of activities that the algorithm can classify). The average of this value for the total of subjects in each study is called AUC_avg_. The difference AUC_avg_-AUC will be smallest for the best technique, assuring that the subtracting AUC values are computed for the same mass of activities. Differences were 0.9854 − 0.9609 = 0.0245, for the personalized form of isotropic algorithm, and 0.9006 − 0.8524 = 0.0482, for the non-personalized form. As 0.0482 ÷ 0.0245 = 1.97, the distance to the maximum AUC in the non-personalized form of the isotropic algorithm is almost double that in the personalized isotropic algorithm, confirming that personalization strongly improves sensitivity and specificity for detecting impacts.

The second row of Figure 2 shows the ROC curves of non-personalized (left) *versus* personalized (right) technique, after removing the addressed influences.

### 3.2. Stage II. Optimal Parameter Region

A key issue for the proper performance of the isotropic algorithm is the selection of the optimum threshold values, *A* and *E*. With that goal, we define the optimum parameter space region R_opt_ as the set of algorithm thresholds that give a sensitivity of 100% (*tpr* = 1), and a specificity greater than (1 − α) × 100%, where α is a number in the range (0, 1). The specificity condition can also be written as *fpr* < α. This manner, R_opt_ *is the parameter space set associated with the ROC region* (*tpr =* 1, *fpr* < α)*.*

**Figure 3:**
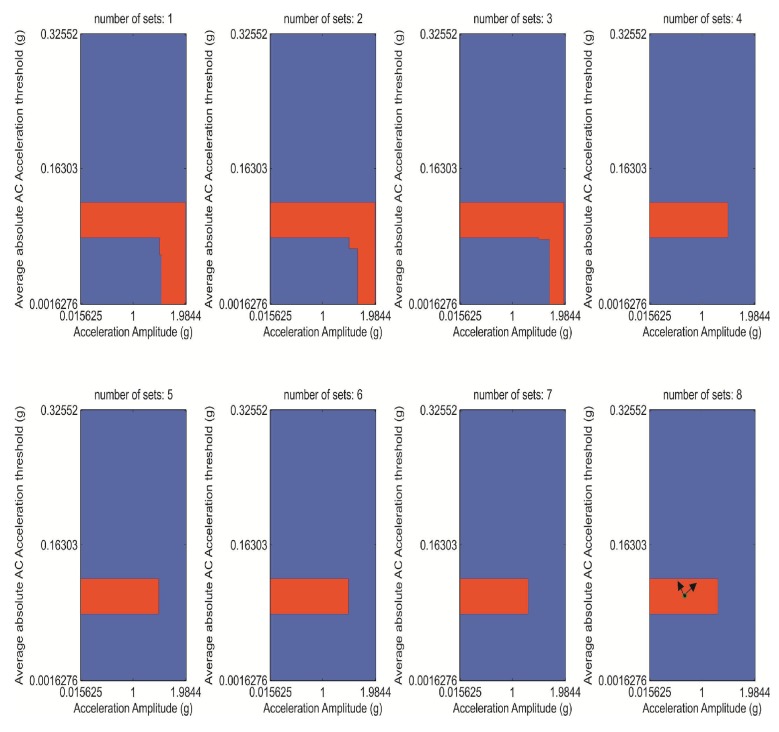
Optimum space region of the personalized isotropic algorithm for α = 0.4, for a growing number of activity sets, from 1 to 8. Threshold values, *E* (vertical) and *A* (horizontal), are written in terms of g units (see text).

When the impact detection algorithm is working in R_opt_, we can guarantee that no impact is missed, whereas the number of false warnings is limited by 100·α%. In addition, if the smart sensor that implements this algorithm operates within a distributed processing architecture, functionally partitioned, then false (and true) positives are really internal events. These events will trigger other types of functional analysis in another computational element, oriented to reduce the final *fpr* value. This concept is the basis of our smart monitor.

As shown in Figure 2, if we define α = 0.4 (specificity >60%), then R_opt_ is empty for the non-personalized form of the isotropic algorithm, because the minimum value of *fpr* for *tpr* = 1 is 0.46. However, the minimum *fpr* for *tpr* = 1 is 0.25 in the personalized form. Therefore, R_opt_ exists under the personalized form of the isotropic algorithm, and is defined by *tpr* = 1 and 0.25 < *fpr* < 0.4.

This region is shown in Figure 3, for a growing number of activities of the user. The convergence of R_opt_, towards a constant region from the 4th set of activities demonstrates the consistency of R_opt_.

As energy is computed by a related variable, *E* by means of Equation (2), both threshold parameters, *A* and *E*, can be written in terms of g acceleration units. Accordingly, we use the average absolute AC acceleration *E_avg_* when referring to values of *E* in text and figures. This is related to *E* through:

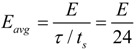
(4)


Dimensions of the R_opt_ in Figure 3 are 0.04 g (*E_avg_*) × 1.5 g (*A*). Acceleration values were coded as 128 counts = 2 g, with a full range of 4 g = 256. Therefore, *A* can be perturbed ±1.5/2 g·128/(2 g) = ±96 counts, and *E* can be perturbed ±0.04/2·24 g·128/(2 g) = 31 counts, from the center of R_opt_, without exiting that region.

We conclude that personalization promotes the existence, consistency, and robustness of R_opt_, with more severe conditions (greater specificity values) than classic non-personalized techniques.

Isolines associated with the objective function *F* = *N*·(3 *tpr* ‒ 1) – *P*·*fpr*, used in a preliminary work [19] are shown in Figure 2. The values (*tpr*, *fpr*) that maximize *F* are (0.986, 0.4) for the non-personalized form of the isotropic algorithm, and (1, 0.25) for the personalized form. Optimum *fpr* and *tpr* values given by this objective function do not guarantee sensitivity values of 100%, and therefore that method for selection of parameters must be discarded. A better method is obtained subsequently.

### 3.3. Stage III. Functional Partition

**Figure 4:**
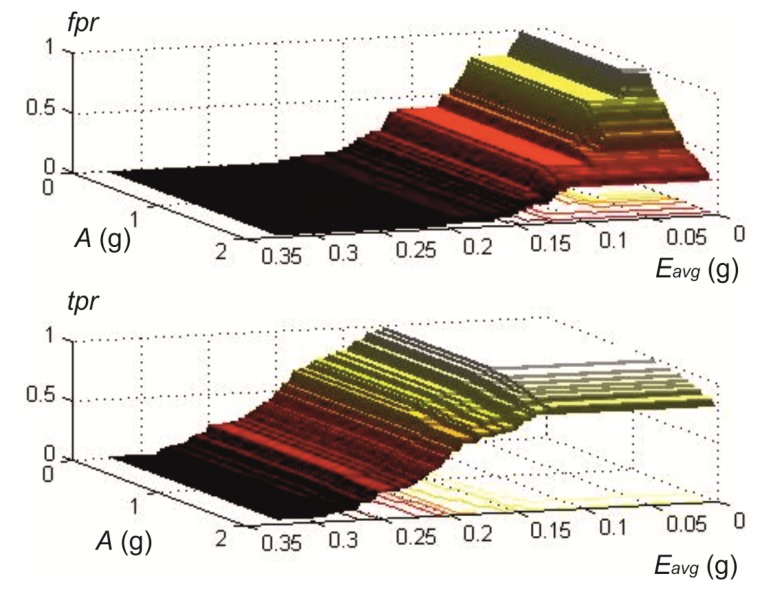
*fpr* (top) and *tpr* (bottom) values as a function of threshold values for the personalized isotropic algorithm.

We have analyzed the influence of *A* and *E* thresholds on *fpr* and *tpr* values. Results for the personalized form of the isotropic algorithm are presented in Figure 4.

As shown, parameters *A* and *E* can be considered decoupled, whereas *E* is the parameter that better governs the sensitivity (*tpr*) and specificity (1 ‒ *fpr*) of the isotropic algorithm. The influence of *A* is very abrupt, and in addition, it does not allow for reducing *fpr* while *tpr* = 1 is kept, in opposition to *E*. This behavior could explain difficulties that present in many current falling detectors (impact-based), founded on the analysis of the amplitude of the acceleration signal, to combine good sensitivity with a low false positive rate.

As a consequence, we can remove the A threshold from Equation (1). This gives the following energy-based isotropic algorithm for impact detection:

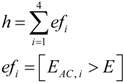
(5)


This new algorithm agrees with the hypothesized functional partition of the computational architecture, where a very light and skin-fixed smart sensor is devoted to the energy analysis for detecting impacts, and a more complex processing device, separate from the body, performs subsequent signal processing focused on body kinematics.

### 3.4. Stage IV. Reachability

The previous stage proves that the energy is the feature that characterizes impact*s*, whereas the instantaneous amplitude does not give useful information about them. This conclusion gives a clue with regard to the way of setting the parameters that optimize detection; that is, to reach R_opt_. We hypothesized that each type of physical activity is associated with a characteristic value of energy for a particular subject, although these values can evolve with the physical state and habits of the subject.

Under that assumption, we can set the *E* threshold as the energy that features a non-impact activity. The *fpr* value will be low enough if this non-impact activity is energetic. This strategy gives a continuous and unsupervised learning method for reaching R_opt_, with the ability to be adapted to the evolution of the subject, provided that impacts can be confirmed in the upper processing layer.

To test the assumption and develop this strategy, we analyzed the energies of the physical activities performed in our laboratory study. The value of the activity’s energy for a particular axis was computed as the mean value of the energy inside the temporal window (*t_i_*, *t_f_*), as follows:

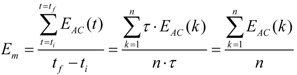
(6)


Initial, *t_i_*, and final time, *t_f_*, are related by *t_f_* = *t_i_* + *n*τ, where *n* is the number of energy sampling times inside the window. The values *t_i_* and *t_f_* were identified for each physical activity as the instants where energy suffers a significant change, with respect to earlier or later values, respectively (*E_AC_* varies e^−1^ per unit in *t_i_* and *t_f_*).

**Figure 5:**
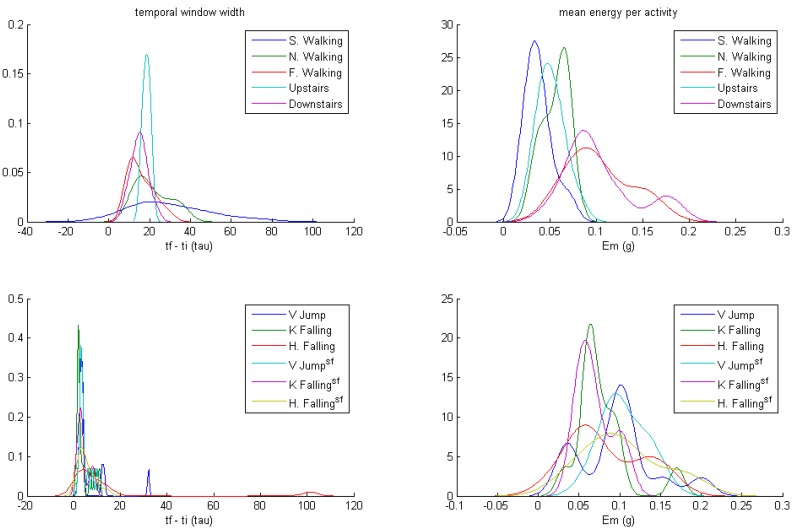
Density plots of temporal window width, and mean energy per type of activity (measured in sensor axis with higher energy), for the non-personalized study. Top: non-impact activities. Bottom: impact activities. ^sf^ Soft floor.

**Figure 6:**
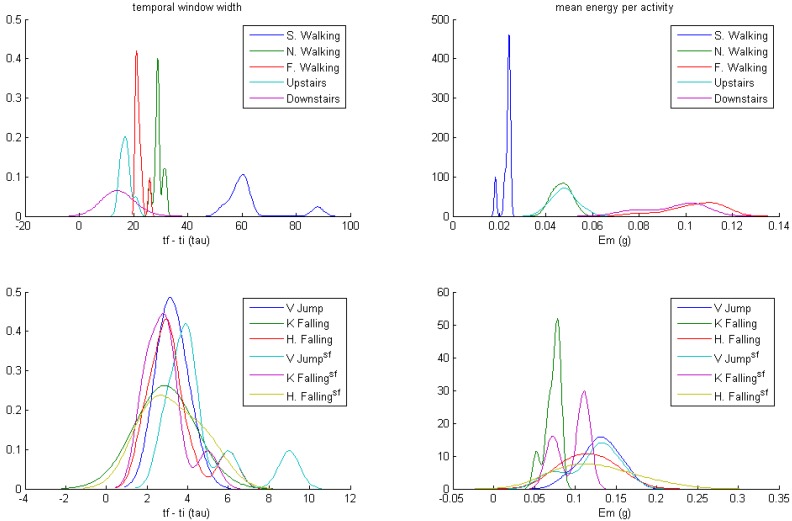
Density plots of temporal window width, and mean energy per type of activity (measured in sensor axis with higher energy), for the personalized study. Top: non-impact activities. Bottom: impact activities. ^sf^ Soft floor.

The density plots of the mean energy, *E_m_*, and the temporal window width, for each activity, are presented in Figure 5 (non-personalized) and Figure 6 (personalized). Activities are shown clustered in non-impact and impact ones. Distributions of temporal window widths for impact activities have much less dispersion in the personalized than the non-personalized study (note the different scale of abscissas). This result agrees with the above hypothesis, concerning the characterization of each type of physical activity in a subject. This difference is less in non-impact activities, because their temporal length is not associated with the subject, but with the length of walk. The subjects were instructed to perform the activities with a high degree of freedom.

Distributions of the mean energy associated with the type of activity do not overlap in the personalized case, in contrast to the non-personalized case, for non-impact activities. In the case of impact activities on a hard floor, despite the overlapping, the energy distributions show a more regular pattern and less dispersion in the personalized case than in the non-personalized. Virtually the same occurs with impact activities on a soft floor, excepting falling to their knees, whose distribution exhibits two significant local maxima. The definition of soft floor impact activities was pursued to increase the difficulty of detecting impacts, because they induce more variability in energies.

These results support the validity of the initial assumption of energies. The main results of this analysis are presented in Table 1.

As expected, standard deviation (SD) of the temporal window width was much less in the personalized than in the non-personalized study. The high value of SD for slow walking is reasonable, since the associated energy is similar to standing up.

Table 1 shows the mean ± SD of each *E_m_* for the total sets of each study. We have selected the axis where mean value was highest, because it is more representative of the energy value for the activity. This axis can be different for each set of physical activities.

**Table 1:** Main results of the energy study. Values are presented as mean ± SD for all sets in each laboratory study: personalized (8 sets, left); and non-personalized (12 sets, right).

*Activity*	*t_f_ − t_i_*	*E_m_*	*E_mmax_^&^*	*E_ACmax_*	*t_f_ − t_i_*	*E_m_*	*E_mmax_*	*E_ACmax_*
Slow walking	62.5 ± 10.7	0.023 ± 0.002	0.025 0.027	0.038	29.6 ± 19.0	0.038 ± 0.014	0.071	0.101
Normal walking	29.1 ± 1.8	0.046 ± 0.004	0.053 0.049	0.082	21.4 ± 8.5	0.056 ± 0.014	0.089	0.142
Fast walking	22.1 ± 1.7	0.103 ± 0.012	**0.115** **0.090**	**0.200**	15.8 ± 6.3	0.102 ± 0.033	0.160	0.252
Going upstairs	17.1 ± 1.9	0.048 ± 0.005	0.057 0.043	0.081	18.4 ± 1.7	0.051 ± 0.014	0.092	0.127
Going downstairs	14.4 ± 2.9	0.094 ± 0.012	0.108 0.072	0.191	15.0 ± 4.1	0.103 ± 0.039	0.189	0.312
Vertical jump	3.2 ± 0.4	0.130 ± 0.022	0.160	0.257	7.2 ± 8.6	0.097 ± 0.048	0.200	0.298
Knee falling	2.9 ± 0.6	0.072 ± 0.010	0.081	0.163	3.9 ± 3.3	0.078 ± 0.034	0.170	0.239
Horizontal falling	3.2 ± 1.3	0.115 ± 0.026	0.160	0.284	14.2 ± 27.7	0.083 ± 0.046	0.162	0.315
Vertical jump ^sf^	4.6 ± 2.0	0.121 ± 0.032	0.163	0.264	4.4 ± 2.4	0.0105 ± 0.027	0.162	0.282
Knee falling ^sf^	2.9 ± 1.0	0.096 ± 0.020	0.115	0.203	3.9 ± 2.6	0.069 ± 0.021	0.158	0.189
Horizontal falling ^sf^	3.2 ± 1.3	0.125 ± 0.043	0.203	0.373	5.4 ± 3.7	0.103 ± 0.048	0.186	0.380

^sf^ Soft floor. Time dimension is units; ^&^ Two values of *E_mmax_* are given for the personalized case and non-impact activities (see text); Energy dimension are in g units (according to *E_avg_* scale).

Table 1 presents the maximum *E_m_*, calculated using Equation (6) for each activity, *E_mmax_*, together with the maximum sampled energy, calculated with Equation (2), for each activity (*E_ACmax_*). *E_ACmax_* is the highest value of *E_AC_* measured for a particular activity, among all executions and axes. As shown, this value is much greater than the associated *E_mmax_*. That is, sampled energies oscillate greatly along the execution of a type of activity, and then we cannot use *E_AC_* to characterize the type of activity. However, we can set the energy threshold as the maximum *E_m_* value obtained by an unsupervised and continuous learning method, for the most energetic non-impact activity. The method is given by the following incremental equation, derived from Equation (6):

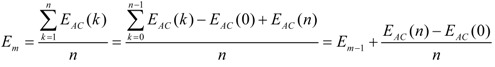
(7)


**Figure 7:**
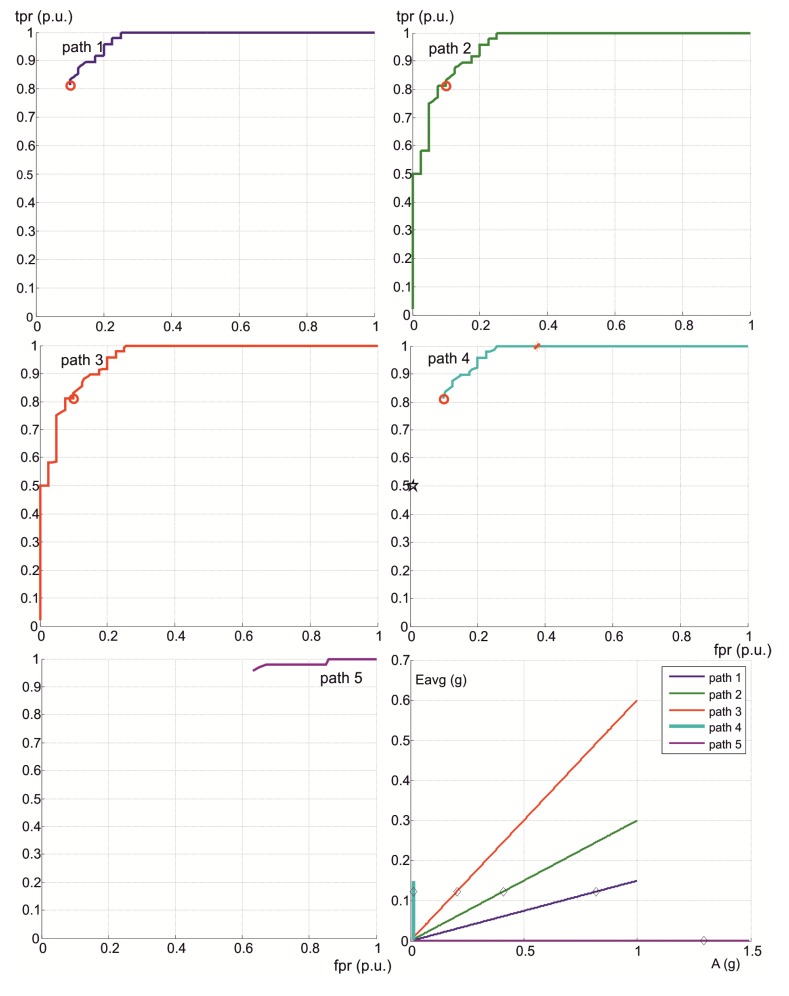
ROC curves associated with the paths of thresholds A–E in the parameter’s space, for the personalized isotropic algorithm with 4 axes. Diamond points in the parameter space refer to threshold values, where the associated ROC curve quits the *tpr* = 1 line. Red circles on the ROC curves correspond to *E* = 0.15 g (*E_avg_* scale) for each parameter’s space path.

In our study, the maximum value of *E_m_* occurred for fast walking (*E_mmax_* = 0.115 g; see Table 1). To test the reliability of this technique in the personalized isotropic algorithm with 4 axes, we computed the ROC points associated with several thresholds’ trajectories in the parameter space. Results are presented in Figure 7.

Red circle points on ROC curves match *E* = 0.15 for each path in the parameter space. They have been used as a reference to check the null influence of *A*. Diamond points in the parameter’s space match points where ROC curves quit the *tpr* = 1 line (and thus the R_opt_ region). Except in the horizontal path (*E* = 0), the value of E was 0.123 g for all paths. Path 4, which is the projection of the remaining paths on the *E* axis, is able to control the reachability of R_opt_, confirming the possibility of withdrawing *A* from the impact detection algorithm.

Moreover, path 5 shows the bad behavior of an impact detection algorithm, based only on the amplitude of acceleration signals. The associated ROC curve keeps a sensitivity of 100% (*tpr* = 1) provided the false positive rate is greater than 0.85.

The behavior of the learning method can be analyzed in path 4, on account to the null influence of *A*. The ROC curve associated with path 4 marks the point matched with the threshold *E = E_mmax_* = 0.115 g, obtained by the learning method using Equation (7), with a red asterisk (*fpr* = 0.37, *tpr* =1). This is a conservative position inside R_opt_ (α = 0.4). The ROC point associated with the *E_ACmax_* value for non-impact activities (*E = E_ACmax_* = 0.200) is shown with a black star point (*fpr* = 0.01, *tpr* = 0.5), which confirms that sampled energies are not a good choice to define the algorithm’s thresholds.

The implementation of Equation (7) implies the computation of n, detecting significant changes in E_AC_, as indicated previously. Although this calculation requires low processing capacity, Table 1 shows a second value of *E_mmax_* for non-impact activities, obtained with the constant value n = 30 (approximately 10 times the highest SD). The resulting threshold (maximum *E_m_* for non-impact activities) is *E* = 0.090 g, which is less than 0.123 g, and pertains to R_opt_. The method for obtaining the threshold by means of Equation (7) is able to learn from the subject, following an unsupervised and continuous method, since *E_m_* values are changing values.

### 3.5. Stage V. Anisotropic Algorithm

The use of four axes in the IAU was justified mainly by the availability to detect errors in the acquisition of signals, and in a second term by the cost and availability of 2-axis sensors against 3-axis sensors. The reduction of prices and the current availability of 3-axes solutions with an autochecking system have pushed us to reduce from four axes to three axes. We have analyzed the behavior of the adaptive isotropic algorithm, after withdrawing the 4th axis (bisectrix of vertical and horizontal axes, into sagittal plane).

As expected, the AUC value for three axes was 0.9581, which is smaller than the AUC value for 4 axes, 0.9609. To improve the AUC we have modified the algorithm given by Equation (5), assigning anisotropy to the impact event condition, as follows:

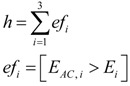
(8)


**Figure 8:**
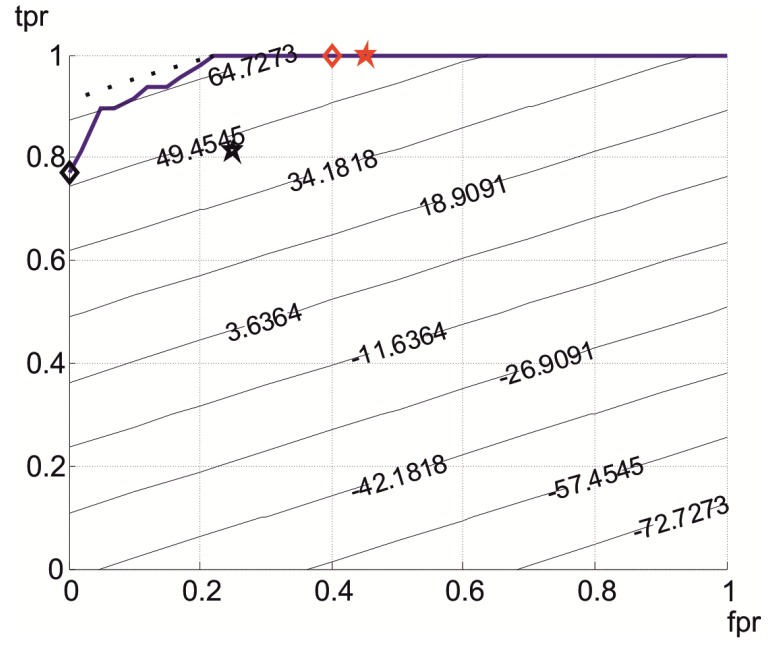
Enveloping curve of ROC points, associated with impact detection in the anisotropic algorithm for the personalized laboratory study. Isolines of *F* are presented to complete the comparison with Figure 2.

This energy-based anisotropic algorithm for impact detection only uses three independent measurement axes, *i* = 1, 2, 3. We tested it in our laboratory study. Figure 8 shows the enveloping ROC curve obtained for the anisotropic algorithm (Equation (8)) in the personalized study.

The AUC value of the adaptive anisotropic algorithm associated with the ROC curve of Figure 8 was 0.9810, greater than the AUC of the adaptive isotropic algorithm for three axes, 0.9581, and for four axes, 0.9609. Moreover, the minimum value of *fpr* has decreased from 0.25 (4-axis adaptive isotropic) to 0.22. These results confirm the superiority of the anisotropic algorithm.

The anisotropic algorithm requires the extension of the unsupervised continuous learning method. This was done by setting the vectorial threshold (*E_1_*, *E_2_*, *E_3_*) as the highest values reached in each axis for non-impact activities. This method was applied to the personalized laboratory study, giving (0.1197 g, 0.0513 g, 0.1085 g) for the vectorial threshold. Temporal windows of activities were similar to those obtained in Table 1, although they changed slightly because of the removal of the bisectrix axis. The operation point associated with this threshold is shown in Figure 8, by means of a red diamond (*fpr* = 0.4, *tpr* = 1), which confirms the robustness and reliability of this unsupervised learning method. Similarly, Figure 8 also presents the point associated with the *E_ACmax_* value for non-impact activities (0.2005 g, 0.0918 g, 0.1908 g), shown by a black diamond (*fpr* = 0, *tpr* = 0.77).

We tested the previous method against another extension, which sets the vectorial threshold as the vectorial energy whose magnitude is highest. This method gives (0.1197 g, 0.0513 g, 0.0806 g) for the threshold, and (0.2005 g, 0.0827 g, 0.1273 g) for the vectorial *E_ACmax_*, which are shown in Figure 8 as red star and black star points, respectively. These results suggest that the operation point can be reached more efficiently addressing the energy of each axis independently, as is done in the learning method selected. In addition, this learning method does not need to compute the magnitude of a vector, and is more efficient computationally. The study has also shown that the R_opt_ associated with the anisotropic algorithm is consistent and robust.

### 3.6. Adaptive “Divide and Conquer” Smart Monitor (DCSM)

**Figure 9:**
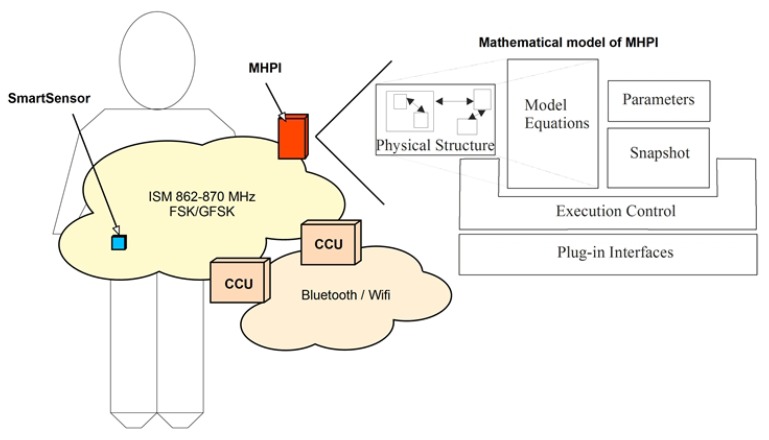
Block diagram of the Divide and Conquer Smart Monitor (DCSM), showing a smart sensor that is connected by ISM–FSK/GFSK wireless technology to the mobile-HPI (MHPI), as well as to several Communication Converter Units (CCU).

The above results support the design of a novel smart monitor for physical risk events, based on a “divide and conquer” strategy (DCSM). A basic block diagram of this system is presented in Figure 9.

The DCSM is constituted by Smart Sensors (SS) that are worn by the subject in a discrete and comfortable manner, linked to a central signal-processing device that is called a Mobile-HPI (Human Physiological Image). The SSs implement the adaptive anisotropic impact detection algorithm, and send impact warnings to the Mobile-HPI, which, in turn, performs a second processing analysis to know the subject activity, and sends back the result (PRE confirmation) to the SS. the Mobile-HPI performs periodic analysis, untriggered by the SSs, to detect non-impact-based PREs.

HPI is a technology that has evolved from PPI, oriented to patients [28], to a generalized form for humans [29]. In the case of the DCSM, it implements a biomechanical model with a low number of degrees of freedom (DoF). HPI is implemented in a Smartphone, which communicates with the SS through wireless ISM technology, indicated in Figure 9. When DCSM operates in outdoor environments, the SS and Smartphone are worn by the subject, and communicate directly. When DCSM operates indoors, the Smartphone communicates with the SS via Communications Converter Units (CCUs), in such a way that the MHPI can be placed in its cradle. At the end of 2010, this architecture was presented as a preliminary work at an international meeting [20], and is patent-pending.

## 4. Discussion

This paper presents an algorithm to detect body impacts with the ability to attend to the changing characteristics of the subject under surveillance. Our study has demonstrated that the very light, adaptive energy-based impact-detection anisotropic algorithm, given by Equation (8), can provide a sensitivity equal to 100%, with values of specificity up to 78%, under the conditions of the study. Its capability to separate impact from non-impact activities is equal to 98.10% (AUC = 0.9810). These results have been based on a total of 20 sets of activities, separated into 12 and eight sets (non-personalized *vs*. personalized study), with 13 healthy volunteers. During the analysis of the algorithm, we have also verified that the optimum region of the algorithm’s parameters is *consistent*, *robust*, and *reachable* by an unsupervised and continuous learning technique, that can be implemented with a very low processor load.

The good performance of the adaptive anisotropic energy-based algorithm supports the methodological issues defined in the methods section (personalization, PRE detection, division of functions, and ability to attend user preferences), which, in turn, are the basis of a “divide and conquer” smart monitor (DCSM), focused on the detection of impact-based PREs, and non-impact-based PREs. The complete definition of the DCSM exceeds the scope of the paper. However, a sketch of its architectural design has been presented for a better comprehension of the algorithm’s context. Our study has also suggested the reasons why impact-based fall-detector devices, founded on amplitude thresholds, have low reliability and a high false positive rate.

Recent progress in wearable falling detectors takes into account mobile technologies and advances in microelectronics, but the adaptive approach has not been exploited until now, to the best knowledge of the authors [30,31,32]. We have selected two relevant impact-based falling systems, in order to compare methods and results. The study from Bourke *et al*. achieved a value of 100% for sensitivity and specificity, by means of an algorithm based on angular velocities, measured on trunk by a bi-axial gyroscope, during a laboratory study on 10 young volunteers [33]. However, they compared fall events from young people against ADL (activities of daily living) from older people. As a consequence, their methodology made the classification of falls and non-falls easier. In addition, their algorithm requires sophisticated filtering, and integral and derivative functions, which were performed off-line with the data acquired by a portable data-logger.

The recent thesis from Kangas addresses fall detection by means of a triaxial accelerometric device [34]. The author performed a very complete study of impact-based fall-detection algorithms, in a laboratory environment. Her results supported the concept of fall detection by means of a waist-worn 3D accelerometer. However, it required the horizontal end posture to define the falling. She pointed out that the use of a simple threshold for impact detection does not provide good performance, in opposition to the results of Bourke *et al*. The discrepancy with Bourke et al.’s results (100% sensitivity and specificity) is justified in Kangas’s work by the fact that Bourke uses young subjects for impacts and older subjects for normal (non-impact) activities, in agreement with our above comment. Fall-detection sensitivity and specificity for a waist-worn accelerometer, from three different scenarios under intentional falls in the laboratory, were 97% and 100%, respectively.

We think that our study has thrown light on the problem of using thresholds for impact detection by 3D accelerometers, besides providing an adaptive algorithm and a methodology to analyze the goodness of detection.

Following the development and testing in laboratory conditions of the novel impact detection algorithm, we are building laboratory prototypes of the DCSM, and adding studies where the smart sensor is placed in other body positions. Preliminary results are successful, showing that even the wrist is a reliable position under our adaptive approach, provided that sampling frequency is increased to attend the frequency spectrum of wrist accelerations. Wrist position has been addressed in Kangas’s work, with poor results. However, details exceed the scope of this work.

We will perform real-life studies in subsequent phases. However, the laboratory study presented in this work is necessary to validate the algorithm and methodology. Recent studies that advance the evaluation of fall detection, under real-life conditions, confirm the validity of intentional (laboratory) falls of younger subjects as surrogates for the real-life falls of older people, in development phases [34]. Despite this support, one of the major limitations of the present paper is the design of the study for laboratory conditions and younger subjects.

## 5. Conclusions

The work has successfully presented and tested an adaptive algorithm, to detect impact-based falls. Results suggest that it could support the development of the novel device, DCSM, able to fulfill both the reliability and ergonomic features required by physical-risk-event monitors.
